# 

**Published:** 1895-03-23

**Authors:** 


**Notices of Births, Deaths, and Marriages** are inserted in this column at a charge of 2s. 6d. for an announcement not exceeding four lines.

**Notice to Advertisers and Subscribers.**

All Advertisements, Orders for Copies of The Hospital and Business Communications should be addressed to **The Manager, NOT TO THE EDITOR,** The Hospital, 428, Strand, London, W.C.

The Cost of Subscription, post free, is as follows:—Per week, 2½d.; per quarter, 2s. 8d.; per half-year, 5s. 3d.; per annum, 10s. 6d. Foreign:—Per Annum, 15s. 2d.; payable, in all cases, in advance.

### NOTICE TO CORRESPONDENTS.

All MS., Letters, Books for Review, and other matters intended for the Editor should be addressed The Editor, The Lodge, Porchester Square, London, W.

The Editor cannot undertake to return rejected MS., even when accompanied by stamped directed envelope.

**"Burdett's Hospital Annual," 1895,**

Ready Immediately.

Sixth year of publication. About 1000 pp. Scarlet cloth, gilt lettered. Price 5s. A most complete guide to British, Colonial, Indian, and American Hospitals, Dispensaries, Nursing and Convalescent Institutions, and Asylums. A few copies of the "Annual" for 1894 may still be obtained on application to the Publishers, The Scientific Press (Limited), 428, Strand, London, W.C.

**NOTICE.—The Index for Vol. XVI. (ending September 24th, 1894) is on sale at the Office of this Journal, Price 2½d., post free.**

### SCRAPS AND GLEANINGS.

By his will Mr. Rawdon has left the sum of £2,000 to the York Dispensary.

It has been proposed to urge a license law for chiropodists in New York State.

The Queen has contributed £25 to the fund for the extension work at the Miller Hospital, Greenwich, of which Her Majesty is patron.

About £52,000 has already been promised towards the proposed new infirmary at Paisley, which is designed for the accommodation of 150 patients.

Extra rooms are going to be furnished and beds provided in extension of the accommodation provided at the Beau Site Convalescent Home, Hastings.

The new wing of the West Ham Hospital, which has been just built through the generosity of Mr. J. Passmore Edwards, at a cost of £3,800, will be formally opened on April 20th.

According to the Registrar-General's weekly return, the deaths attributed directly to influenza last week amounted to 473, showing some considerable increase in the numbers of the previous weeks.

An increase of patients for the past year is recorded at the Liverpool Cancer and Skin Diseases Hospital, an average attendance of nearly 100 a day being entered in the books of the institution.

Last week the Plowden Bijou Orchestra, a society of amateur musicians, of which Mr. Santley is conductor, gave a concert at the Brompton Hospital for Consumption for the entertainment of the patients.

The Bideford and District Dispensary and Infirmary is successful in carrying forward a favourable balance on this year's accounts. The institution is one doing a useful work, and extending over an exceptionally large area.

A cottage hospital (twenty beds) will be opened at Loughton, Essex, in April. A limited number of cancer patients will be admitted. No letters of recommendation will be needed, though suitable cases only will be admitted of both sexes.

The Duke and Duchess of York visited St. Mary's Hospital, Paddington, yesterday. Their Royal Highnesses arrived at three p.m. and were conducted through the wards by Sir William Broadbent, Bart., senior physician to the hospital.

A Cottage Hospital has been proposed for Malton (Yorks), Norton, and the district. Mr. John Grant Lawson, M.P., in consideration of how great is the need of such an institution, has promised £25 towards the furtherance of the scheme.

A contract has been entered into for the superstructure and corridors to connect a new block with the existing building of the West London Hospital, the cost of which extension will be £14,944. This addition will permit of 80 additional beds.

The annual meeting of governors and subscribers to the Northern Counties Hospital for Consumption and Diseases of the Chest has recently been held. The financial position of the institution, considering the depression in trade, is a satisfactory one. Later on, it is hoped to extend the premises, which are not now of sufficient proportions to cope with the increasing numbers of in-patients.

The question as to what area the proposed Infectious Fever Hospital at Malvern shall include continues unanswered. It is of vital importance that Malvern shall have further infectious diseases accommodation without delay. If the opposition as to area continues to be further urged the Malvern board are prepared to erect a hospital for their own local benefit. Lady Emily Foley has declared herself prepared to give a site for the use of Malvern alone, but not for a joint board.

### THE STUDENT OF MEDICINE.

### APPOINTMENTS.

W. L. Bell, M.D.Edin., appointed Surgeon to the Lowestoft Hospital. R. Bevan, L.R.C.P.Lond., M.R.C.S.Eng., reappointed Medical Officer of Health for Lydd. J. W. Burdwood, L.F.P.S., Glasg., L.M., L.S.A.Lond., Fell. Brit. Inst. Pub. Health, Mem. San. Inst., reappointed Medical Officer of Health for the Bourne Rural Sanitary District. T. Callaghan, L.R.C.P., L.R.C.S.Edin., reappointed Extraordinary Physician to the Cork Fever Hospital. W. Clegg, M.R.C.S.Eng., L.S.A., reappointed Medical Officer of Health to the Boston Rural District Council. J. G. E. Colby, D.P.H.Camb., reappointed Medical Officer of Health for the Malton Rural Sanitary District and the Norton Rural Sanitary District. J. B. Dreaper, L.R.C.P.Edin., M.R.C.S.Eng., appointed Medical Officer of Health to the Ashbourne Rural District Council. F. I. Flower, M.R.C.S.Eng., L.S.A., reappointed Medical Officer of Health to the Warminster Rural District Council. D. J. Flynn, M.D.R.U.I., L.M.R.C.P.I., reappointed Physician to the Cork Fever Hospital. F. W. Gange, M.R.C.S., L.R.C.P.Lond., reappointed Medical Officer of Health to the Faversham Rural District Council. E. Gaylor, L.R.C.P. Edin., L.F.P.S.Glasg., reappointed Medical Officer of Health to the Alfreton Urban District Council. J. A. R. Glennie, M.B. Aberd., appointed Physician to the Aberdeen Dispensary, Vaccine, and Lying-in Institution. F. Gotch, B.A.Lond., B.Sc., M.A. Oxon, M.R.C.S., appointed Waynflete Professor of Physiology at Oxford University. Dr. Haworth, appointed Medical Officer of Health for Darwen. S. Hillier, M.D., appointed Medical Officer for the Stowmarket District of the Stow Union. T. H. Jones, M.B., C.M.Edin., appointed Senior House Surgeon to the Infirmary for Children, Liverpool. C. M. Kempe, M.R.C.S.Eng., L.S.A., reappointed Medical Officer of Health to the Shoreham Urban District Council. H. M. Langdale, M.R.C.S.Eng., L.S.A., reappointed Medical Officer for the Workhouse of the Uckfield Union. H. Laver, M.R.C.S.Eng., L.S.A., reappointed Surgeon to the Essex and Colchester Hospital. A. Mackintosh, M.D. Glasg., L.F.P.S., reappointed Medical Officer of Health to the Clay Cross District Council. J. T. R. Miller, L.S.A.Lond., reappointed Medical Officer and Public Vaccinator for the Leavening District of the Malton Union. G. H. Milnes, B.A., M.B. Cantab., M.D., L.R.C.P., appointed Physician to the Derbyshire Infirmary. T. B. Moriarty, A.B.R.U.I., M.D., L.R.C.S.Edin., reappointed Physician to the Cork Fever Hospital. D. Murphy, M.B., B.Ch., B.A.O.R.U.I., reappointed Resident Medical Officer to the Cork Fever Hospital. J. E. Murphy, L.D.S.R.C.S.I., appointed Consulting Dental Surgeon to the Derbyshire Infirmary. R. D. Prichard, L.R.C.P.Edin., L.R.C.S.Edin., L.F.P.S.Glasg., appointed Medical Officer and Public Vaccinator for the Second Central District of Neath Union. R. T. Richardson, M.R.C.S.Eng., F.Br.Inst.P.H., reappointed Medical Officer of Health for Trowbridge, Wilts. J. Robertson, M.D. Edin., reappointed Medical Officer of Health to the Cockermouth Urban District Council. D. Seaton, M.B., Ch.B.Vict., appointed Resident Casualty Officer to the General Infirmary, Leeds. S. Skinner, M.B.Aberd., appointed Medical Officer to the Clevedon District Council. E. Worts, L.R.C.P.Lond., M.R.C.S.Eng., reappointed Surgeon to the Essex and Colchester General Hospital. J. L. Wright, L.R.C.P.Edin., M.R.C.S.Eng., appointed Surgeon to the Derbyshire Infirmary.

### VACANCIES.

The following vacancies are announced this week, the amount of salary in each case being given after the name of the institution:

### Resident Medical Officers.

Bethlem Hospital.—Two Resident Clinical Assistants; doubly qualified. Appointment for six months. Will be provided with such apartments, rations, washing, and attendance as the Committee shall consider reasonable. Applications, endorsed "Clinical Assistantship," to the Treasurer, at Bethlem Hospital, before March 31st.

Bolton Infirmary and Dispensary.—Junior House Surgeon; doubly qualified; age not to exceed 25. Salary £80 per annum, with furnished apartments, board, and attendance. Applications to Mr. Peter Kevan, Honorary Secretary, 12, Acresfield, Bolton, by March 26th.

City of Glasgow District Lunacy Board.—Medical Superintendent of the new Asylum at Gartloch. Salary at the rate of £450 per annum, with free house, coal, gas, &c. Applications and testimonials to Mr. Dempster, Clerk to the Board, 318, Parliamentary Road, Glasgow, by March 25th.

General Hospital, Birmingham.—Two Assistant House Surgeons. Must possess surgical qualification and be registered. Appointment for six months. No salary, but residence, board, and washing provided. Applications and testimonials, with certificate of registration, to Howard J. Collins, House Governor, by March 30th.

THE STUDENT OF MEDICINE—*continued.*

Glamorgan County Asylum, Bridgend.—Junior Assistant Medical Officer; unmarried, and not over 30 years of age. Salary £100 per annum, with board (no beer or wine), lodging, washing, and attendance. Applications to the Medical Superintendent by March 26th.

Horton Infirmary, Banbury.—House Surgeon and Dispenser; duly qualified. Must be M.R.C.S.Eng., and registered. Salary £60 per annum, with board and lodging. Applications and recent testimonials to Mr. C. H. Davids, Honorary Secretary, 21, Marlborough Road, Banbury, by March 23rd.

London School of Medicine for Women.—Demonstrator of Physiology. Applications to the Secretary, 30, Handel Street, Brunswick Square, W.C., by March 31st.

New Hospital for Women, 144, Euston Road, N.W.—Clinical Assistant for the Out-patient Department, and Assistant Anæsthetist. Appointment for one year. Applications to the Secretary by March 27th.

Radcliffe Infirmary, Oxford.—Honorary Physician. Candidates must be legally qualified to practice as Physician in England. Testimonials to A. C. Virgo, Secretary, by March 25th.

Royal College of Physicians.—Milroy Lecturer. Applications to the Registrar, Royal College of Physicians, Pall Mall East, S.W., by April 9th.

Royal United Hospital, Bath.—House Surgeon, must be M.R.C.S.Eng. Salary £60 per annum, with board, lodging, and washing. Appointment for six months. Applications to W. Stockwell, Secretary-Superintendent, by March 20th.

St. Thomas's Hospital Medical School.—Demonstrator of Physics and Chemistry. Stipend £100 per annum. Applications to the Medical Secretary, St. Thomas's Hospital, S.E., by March 25th.

Taunton and Somerset Hospital.—House Surgeon; doubly qualified. Appointment for three years. Salary £100 per annum, with board, lodging, and washing. Applications to J. H. Biddulph Pinchard, Secretary, 13, Hammet Street, Taunton, by April 4th.

**Non-Resident Medical Officers.**

Charing Cross Hospital.—Assistant Physician. Applications to Arthur E. Reade, Secretary, by March 30th.

Golborne Urban District Council.—Medical Officer of Health. Salary £30 per annum. Applications, marked "Medical Officer of Health," to Charles S. Bennie, Clerk, by March 26th.

Hospital for Sick Children, Great Ormond Street, Bloomsbury, W.C.—Medical Registrar and Pathologist. Appointment for one year. Honorarium 50 guineas. Applications to the Secretary by March 26th.

London Hospital, Whitechapel, E.—Surgical Registrar, from April 1st. Salary £100 per annum. Applications and testimonials to G. Q. Roberts, House Governor, by March 29th.

Westminster Hospital, Broad Sanctuary, S.W.—Medical Registrar; must be a legally qualified medical practitioner. Appointment for twelve month. Salary £40 per annum. Applications to Sidney M. Quennell, Secretary, by March 25th.

### UNIVERSITIES AND COLLEGES.

### ROYAL COLLEGE OF SURGEONS IN IRELAND.

Fellowship Examination.—The following gentlemen, having passed the necessary examination, have been admitted Fellows of the College: R. B. McCausland, G. R. Penrose, and H. Stoker.

Anatomical Prizes for Students.—The Royal College of Surgeons in Ireland announces that two Barker Anatomical Prizes of the value of twenty-five guineas each are offered for competition this year, and are open to any student whose name is on the anatomical class list of any school in the United Kingdom. One prize will be allotted to a dissection of the kidneys as seen from behind, special value being attached to the exhibition of the surgical relations. The other prize will be given for a dissection of the rectum as seen from behind, special value being attached to the exhibition of the relations of the bowel in connection with the operation of removing it by the trans-sacral method. The preparations must be received by the Curator of the Museum before June 1st, 1895, and from him full particulars can be obtained.

### SOCIETY OF APOTHECARIES OF LONDON.

Pass List, February, 1895.

The following candidates passed in:

Surgery.—L. M. Breton, St. Thomas's; W. J. Gillespie, St. Bartholomew's; D. W. Jones, Charing Cross; S. E. Price, London; H. Roberts, St. Mary's; W. Sutcliffe, Birmingham; and M. M. Townsend, King's.

Medicine, Forensic Medicine, and Midwifery.—A. F. Blake, London; W. Mettam, Sheffield; and H. H. Thomas, Charing Cross.

Medicine and Forensic Medicine.—W. E. Bremner, King's; and R. Keatinge, London.

Medicine and Midwifery.—H. G. C. Hardwick, St. Thomas's.

Medicine.—H. Roberts, St. Mary's.

Forensic Medicine and Midwifery.—S. S. Wallis, Guy's.

Forensic Medicine.—J. C. G. Reed, Guy's; and G. Lowsley, St. Bartholomew's.

Midwifery.—A. H. Wade, St. Bartholomew's.

To Messrs. Jones, Keatinge, Mettam, Price, Roberts, and Sutcliffe was granted the diploma of the Society, entitling them to practise Midwifery, Surgery, and Midwifery.

